# Supplementary material for: Complexome profiling of the Chlamydomonas psb28 mutant reveals TEF5 as an early PSII assembly factor
Source: Plant Cell. 2025 Mar 17;37(6):koaf055. doi: 10.1093/plcell/koaf055 (PMC12164590; doi:10.1093/plcell/koaf055)
Supplement: koaf055_Supplementary_Data [file koaf055_supplementary_data.zip › psb28_interactive.zip!/plots/intensity/qualityPlots/peptides/_Cre09.g393358.t1.1.html]

### Cre09.g393358.t1.1

Trivial name:   
SeqCoverage [median]: 7.80  
Mass: 37.54 kDa  
  
 

| Sequence | MissedCleavage | Mass [Da] | wtIntensity | muIntensity | Score |
| --- | --- | --- | --- | --- | --- |
| EAPKEEAKPAKAPVAK | 3 | 1662.93 | 659.28 | 0.00 | 54.81 |
| NVDPNAELKGLK | 1 | 1296.70 | 634.84 | 0.00 | 37.61 |
|  |  |  | 1294.1 | 0.0 |  |
